# Graphene/Glycerin Solution-Based Multifunctional Stretchable Strain Sensor with Ultra-High Stretchability, Stability, and Sensitivity

**DOI:** 10.3390/nano9040617

**Published:** 2019-04-16

**Authors:** Zhenkun Qi, Hailiang Bian, Yi Yang, Nantian Nie, Fuliang Wang

**Affiliations:** School of Mechanical and Electrical Engineering, Central South University, Changsha 410083, China; qizhenkun@csu.edu.cn (Z.Q.); csucmeebhl@csu.edu.cn (H.B.); youngwhyi_csu@163.com (Y.Y.); nienantian@csu.edu.cn (N.N.)

**Keywords:** stretchable sensor, conductive liquid, graphene, glycerol, motion detect

## Abstract

Highly stretchable, flexible, and sensitive strain sensors have promising applications in motion detection—especially multifunctional strain sensors that can detect stretching, bending, compression and twisting. Herein, this study presents a graphene and glycerol solution-based multifunctional sensor with ultra-high stretchability and sensitivity. Owing to the self-lubrication and fluidity of the graphene-glycerol solution, the strain sensors display super stretchability up to 1000%, a maximum gauge factor up to 45.13, and excellent durability for over 10,000 cycles. In addition, the sensor can also rapidly respond to small strains (1%, 5%, 10%) and different stretching rates (12.5%/s, 25%/s, 50%/s, and 100%/s). More impressively, the sensors can measure up to 50 kPa pressure and 180° twisting without any damage. Furthermore, the strain sensors demonstrate their applicability in scenarios involving motion detection, such as that for finger bending, wrist rotating, touching, and drinking water.

## 1. Introduction

Stretchable flexible strain sensors can convert mechanical deformations (strain, pressure, bending, twisting) to electrical signals [1], which can be widely used for motion detection [2] and health monitoring [3,4,5]. However, traditional metallic foil strain gauge sensors are poorly suited to the high sensitivity over small ranges of motion required for these applications.

Recently, research efforts have been devoted to developing flexible strain sensors by combining conductive nanomaterials [6] and flexible substrates [6,7]. Because nanomaterials are smaller and more conductive, they are more suitable for micro or nanoscale motion than metallic foil. In these nanomaterial-based flexible strain sensors, the motion of the flexible substrate can change the contact states of the nano-conductive components and thus change the resistance/conductivity of the sensor. Based on this mechanism, many flexible sensors have been developed using nanoparticles [8,9,10,11], nanowires [7,12,13], CNTs (carbon nanotubes) [14,15,16,17], graphene [2,18,19,20], MoS_2_ [21], and nanocomposites [22,23,24,25]. However, these conductive nanomaterials solidly connect to each other, preventing extra-large deformation and limiting the sensor strain range to 280%.

To overcome this limitation, liquid, conductive, nanomaterial-based flexible sensors were developed, wherein the conductive solution was encapsulated in a flexible substrate, permitting enough deformation for large strains due to the excellent fluidity. Such strain sensors based on reduced graphene oxide/DI (deionized water) sensing liquids [26] and another conductive ionic liquid [27] (1-Butyl-1-methylpyrrolidinium tetracyano borate) have been reported.

However, these nanomaterials in a solvent do not have enough motion resistance, as the viscosity of deionized water is low, making the sensor unstable, especially for large deformations. Therefore, there is still a need to develop a multifunctional strain sensor which can detect strain, bending, pressure, and twisting [28,29].

In this study, we developed a novel graphene/glycerol (G/GL) solution-based, multifunctional, stretchable sensor wherein the graphene was dispersed in the glycerol. Theoretically, the viscous glycerol provides lubrication for the graphene during sensor deformation and motion resistance during sensor recovery. Therefore, it was expected to perform well for extremely large deformations. The mechanism of these strain sensors is the disconnection between graphene flakes. Due to the fluidity of the graphene solution we created a strain sensor with a 1000% stretchability and maximum gauge factor (GF) of 45.13. Moreover, the strain sensor has excellent cycling stability: 10,000 times within the range of 200%, due to the self-lubricating and viscous properties of glycerol. The sensor can also rapidly respond to small strains (1%, 5%, 10%) and different stretching ratios (12.5%/s, 25%/s, 50%/s and 100%/s). The G/GL sensors can also respond to pressure (50 kPa) and twisting (0–180°). Furthermore, these multifunctional, stretchable sensors have been demonstrated for static and dynamic human motion detection and can recognize wrist rotation in our experiments.

## 2. Materials and Methods

### 2.1. Preparation of Viscous Graphene Solution

One gram of graphene (6–10 layers; flake size: 5–50 µm) was added to four grams of glycerol. The viscous solution was ultrasonicated for an hour and stirred for another hour.

### 2.2. Fabrication of G/GL Based Stretchable Strain Sensors

**Figure 1:**
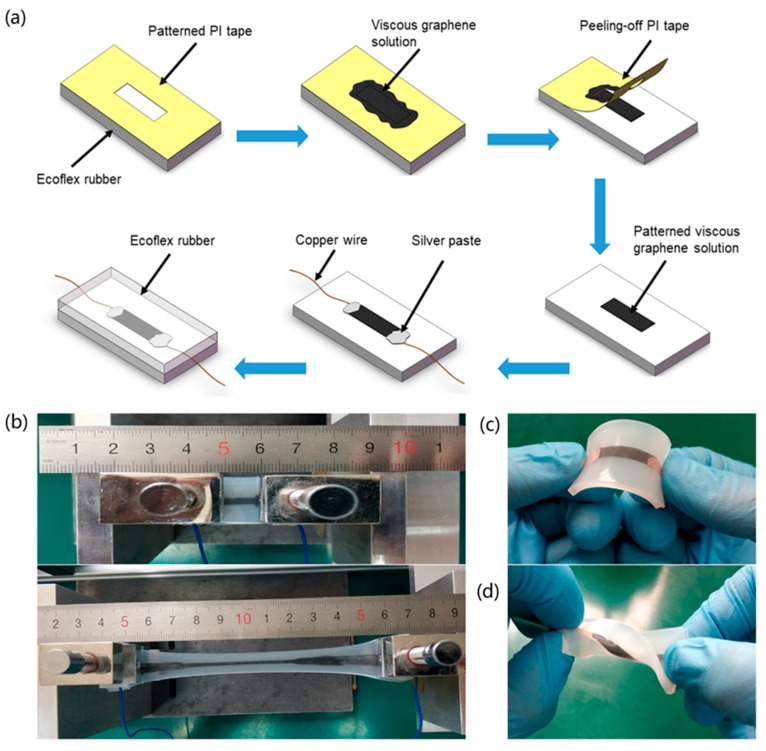
Fabrication process and result of the sandwich-structured Ecoflex rubber and graphene solution strain sensor: (**a**) fabrication process, (**b**) the strain sensor before and after stretching to ε = 1000%, the strain sensor under bending (**c**), and twisting (**d**).

The strain sensor was fabricated using the following procedure (Figure 1a): 20 g Ecoflex rubber (mass ratio A:B = 1:1, Ecoflex00-50, SMOOTH-ON) was poured onto an acrylic mold (length × width × thickness: 40 mm × 20 mm × 5 mm) that had been previously cleaned with acetone, ethanol, and deionized water. After the rubber was cured (1 h at 70°C), the electrode areas were masked on the cured rubber using polyimide (PI) tape. The viscous graphene solution was coated on the exposed rubber, overlapping the PI tape. The PI film was then removed from the Ecoflex rubber, leaving viscous graphene solution on the rubber surface. Copper wires were attached to the ends of the viscous graphene solution with silver paste. Then another layer of liquid Ecoflex was cast over the viscous graphene solution area and the bottom Ecoflex rubber. Finally, the complete part was cured at 70 °C for 2 h.

### 2.3. Sensor Characterization and Motion Detection Experiment

All the concentration 1:4 strain sensor sensing tests were performed on our self-made tester, which is controlled by a stepper motor. The resistance changes were measured and recorded by a digital LCR meter (Applent Instruments Ltd. AT810A, Changzhou, China). In addition, the ends of the strain sensor were wrapped to make sure that the copper interconnection wires were insulated from the metal fixture. When the strain sensor was used to detect body motion, both ends were attached to the test subject’s body using PI tape. The areas tested for motion included the index finger, wrist, forearm, and Adam’s apple. Besides, nine sensors were used to complete all different tests, GFs of these sensors can be seen in Table S1 (Supplementary Materials Table S1).

## 3. Results

### 3.1. Fabrication of Multifunctional G/GL Based Stretchable Sensor

The key fabrication process of the multifunctional G/GL based stretchable sensors is schematically illustrated in Figure 1a. The G/GL based strain sensors can detect stretching, bending, and twisting as shown in Figure 1b–d. Figure 1b shows the fabricated G/GL based sensors being stretched from an initial 1 cm to a final 11 cm, meaning it has stretchability as high as 1000%. Figure 1c,d shows that this multifunctional G/GL sensor also has excellent properties in bending and twisting. The excellent sensing ability of this sensor is mainly due to the viscous and self-lubricated graphene glycerol solution. Because, the G/GL based sensors are completely encapsulated in Ecoflex rubber, they are robust, repeatable, and fit for use on human skin.

### 3.2. Effect of Graphene Concentration in G/GL Solution

**Figure 2:**
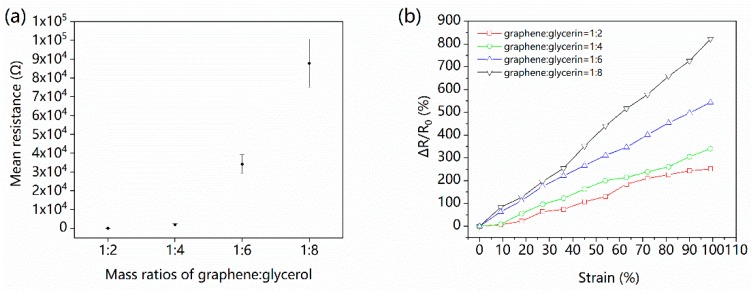
Effects of the graphene:glycerol ratio: (**a**) G/GL solution resistance for different mass ratios; (**b**) relative resistance changes plot of different mass ratios.

The concentration of graphene in the G/GL solution determines two characteristics of the strain sensor: initial resistance and resistance variation (Figure 2). The effect of graphene concentrations on the initial resistance is shown in Figure 2a. With the number of conductive graphene flakes in the solution reduced, resistance increased exponentially. For the lower concentrations of conductive graphene flakes, the average distance between flakes in the solution increases, increasing the initial resistance. Furthermore, G/GL solutions with mass ratios of 1:6 and 1:8 have better fluidity (lower viscosity) than those with mass ratios of 1:2 and 1:4, allowing the graphene flakes to move more easily. This explains the increased standard deviations of the former relative to the latter.

Figure 2b shows the effect of graphene concentration on the resistance variation ratios (RVR) in the strain range of 0–100%. The RVR is defined as ΔR/R_0_ = (R−R_0_)/R_0_, where R and R_0_ are the resistance with strain and without strain, respectively. All samples were stretched at the same strain rate of 25%/s. While stretching to 100%, all RVR curves increase linearly. In the case of G:GL mass ratio 1:2, the RVR of the sensor is 251.7% when the strain is 100%.

The G/GL based sensor with G:GL mass ratio of 1:4 has an RVR value of 341.2% which is higher than the mass ratio 1:2 sensors. The lowest mass ratio sensors (1:8) have the highest RVR value of 822.7%, while the sensor with 1:6 mass ratio has an RVR value of 544.2%. Figure 2b shows that the higher the concentration of graphene, the poorer the motion-detecting ability. A reasonable explanation for these different RVR values is that high mass ratios (1:2 and 1:4) of G/GL solution have higher viscosity and smaller spaces between graphene flakes which make them more difficult to move and have more connections than the low mass ratio (1:6 and 1:8) G/GL solutions.

### 3.3. Stretchability of Sensor under Various Strain Rates, and Durability

**Figure 3:**
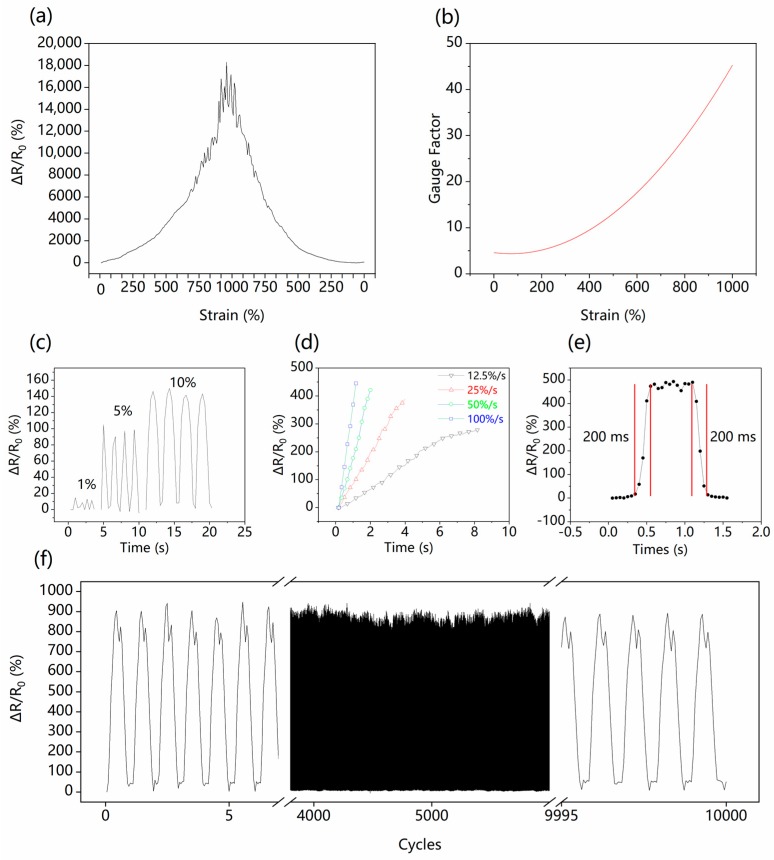
Testing the sensor performance. (**a**) Relative resistance versus strain plot showing stretching to 1000% and releasing, (**b**) gauge factor versus strain from 0–1000%, GFs were calculated by polynomial fitting; (**c**) reversible response of the sensor under small strains from 1% to 10%, (**d**) variation of the resistance of the sensor from 0–100% strain at different strain, (**e**) response/relaxation time of the sensor at a strain of 100%, both of which are 200 ms, (**f**) resistance response of the strain sensor over 10,000 stretch-and-release cycles from 0–200% strain.

We fabricated our G/GL based sensors with G:GL mass ratio of 1:4 for the following tests. The G/GL based sensors display an outstanding stretchability of 1000%, as shown in Figure 1b. To characterize the dynamic response of the sensor, the RVR process during loading and unloading of 1000% strain with a strain rate of 25%/s was recorded, as shown in Figure 3a. In general, the RVR increased exponentially from 0 to 18,283.1% while the strain increased from 0 to 1000%. In the strain range of 0–800%, the RVR increased stably from 0 to 9112.8%. However, in the strain range of 800–1000%, the highly stretched rubber caused larger distances between graphene flakes in the G/GL solution, making the conductive connections between graphene flakes weak and unstable, and causing fluctuation of RVR.

Accordingly, the GF of the sensor was calculated, as shown in Figure 3b. GF is given by (ΔR/R_0_)/ε, where ΔR is the resistance change during stretching, R_0_ is the original resistance as mentioned above, and ε is the applied strain. For low strains (0–100%), the GF stabilized around 4.3. When the applied strain was increased to 1000%, the GF increased to a maximum of 45.13, which means the G/GL based sensors not only have excellent stretchability but also have a high GF at the same time.

Because of the self-lubrication and viscosity of the G/GL solution sensing material, the super-stretchable sensor can also detect small strains. However, its response varies with the stretching speed. The RVR of the sensor for stretch-and-release cycles at small strain levels at a rate of 10%/s was measured, as shown in Figure 3c. At a strain of 1%, RVR of 10% was measured. At 5% and 10% strain, the RVR was about 90% and 140%, respectively.

When the G/GL sensors were stretched to 100% at different rates, the sensors responded linearly, but differently, at each speed (Figure 3d). The higher the stretch speed, the higher RVR can be obtained. For a 100%/s strain rate, the RVR reached 445.34. The reason for this is that the resistance of the G/GL solution is mainly decided by the average distance and amount of connections between graphene flakes. Therefore, the faster the stretching speed, the quicker the separation of flakes, resulting in the quicker increase of RVR. Moreover, as shown in Figure 3e, the G/GL based sensors’ immediate response and recovery time of 200 ms at 100% strain also indicate the excellent dynamic response ability of our sensors. So, the excellent dynamic response ability of the sensor shows good promise for detecting wide-ranges of human motion. 

The long-time cyclic uniaxial tensile test was also performed to demonstrate the repeatability of the G/GL based sensor, shown in Figure 3f. After 10,000 cycles (with 200% stretch) shows that the RVR always varied from 0–900%, and when released, the RVR always returns to 0%, which means the sensor has excellent stability. Our G/GL sensors have a larger GF than other sensors with liquid or solid sensing materials as reported in Table 1.

**Figure 4:**
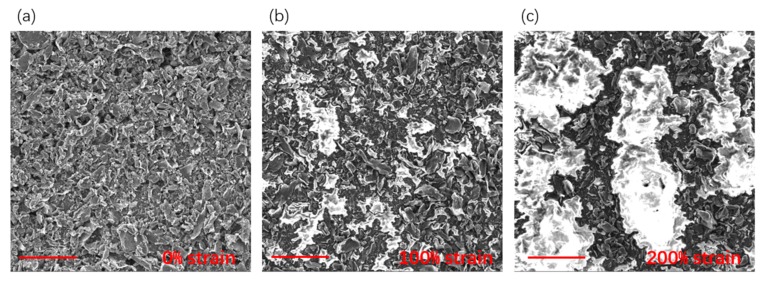
Scanning electron microscopy images of graphene/glycerol sensors without upper-side Ecoflex under 0% (**a**), 100% (**b**) and 200% (**c**) strains, respectively. The scale bars represent 100 μm.

The sensing mechanism of our G/GL sensors is the change of the graphene network. In order to understand the sensing mechanism of the strain sensors, the scanning electron microscopy images (TESCAN VEGA3; 10 kV) of the G/GL sensor without upper-side Ecoflex in different strains are shown in Figure 4. When the sensor was under 0% strain, as shown in Figure 4a, graphene partially connects with each other without separating and splitting. As shown in Figure 4b, when the sensor was stretched to 100%, the graphene began to disintegrate. Once microcracks arose, the overlap between graphene reduced which increased the resistance of the graphene network. When the strain was applied to 200%, the connection between graphene became less and less, as shown in Figure 4c. Meanwhile, adjacent microcracks connected to form a bigger crack, resulting in a larger resistance. This can explain the sensing mechanism of our G/GL sensors is disconnection and connection of sensing material in the liquid, which has also been observed in some previous studies [26,33,39].

### 3.4. Pressure and Twisting Dynamic Response

**Figure 5:**
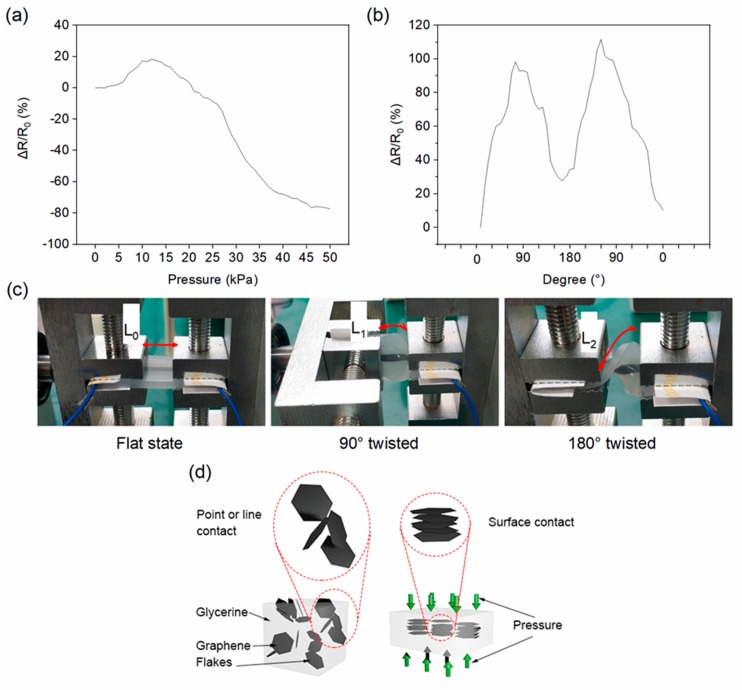
The G/GL based sensor used for pressure and twisting measurements, (**a**) relative resistance variation responses for increasing pressure, (**b**) relative change of resistance versus twisting, (**c**) different states of the sensor during twisting process, (**d**) schematic illustration of the mechanism of the electromechanical responses of the strain sensor under compression.

In addition to stretching, compression and twisting can also be detected with these G/GL based sensors. To measure the pressure detection ability, a thin 20 × 20 mm acrylic plate connected to the stepper motor was placed on the surface of the sensor, and a force gauge was used to monitor the applied pressure. The plate was used to make the applied pressure uniform and stable. Figure 5a shows the RVR changing with the applied pressure from 0 to 50 kPa. In the case of relatively small pressure (≤12.5 kPa), the pressure mainly caused the Ecoflex rubber to expand, which enlarged the horizontal dimensions of the G/GL portion, decreasing the contact between graphene flakes, and resulting in the RVR increasing about 20%. However, when the pressure exceeded 12.5 kPa, the vertical space between graphene flakes decreased, and the flakes reoriented themselves to create face-to-face interconnection, reducing the RVR to −80% when the force was 50 kPa.

In contrast to stretching, compression reduced the distance between graphene flakes and created more contact. Increasing compression turns the point and line contacts of graphene flakes to surface contacts, as shown in Figure 5d. Therefore, compression increases the graphene contact and reduces the resistance of the sensor.

Twisting of the G/GL based sensor was tested with a lab-fabricated torsion tester by twisting the two sides of the sensor and recording the RVR, as shown in Figure 5b–c. When the twisted angle was 90°, the length of the sensor had increased from L_0_ to L_1_. This twist caused stretching and resulted in a 100% increase in RVR. When the twisted angle reached 180°, the length of the sensor was increased to L_2_. However, the stretched bottom-side Ecoflex and compressed upper-side Ecoflex caused compression on the sensing materials similar to applying pressure and decreased the RVR from 100% to 30%. This twisting response can be used for human twisting motion detection.

### 3.5. Application as A Motion Monitor

Since our novel sensor is highly stretchable (up to 1000%) and sensitive (GF as high as 45.13), it can be used for full-range human activity recognition, such as finger bending, wrist rotating, touching, and water drinking.

**Figure 6:**
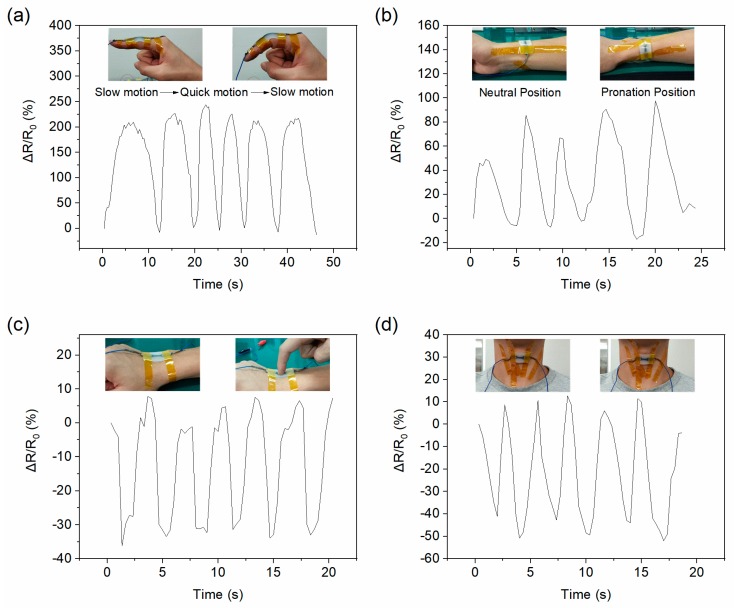
Human body movement detection using the G/GL based sensor; (**a**) finger movement, (**b**) wrist rotation, (**c**) “clicking” the sensor, (**d**) drinking water (movement of Adam’s apple).

The finger bending test was carried out with rapid bending and slow bending, and the sensor responded with different maximum values of RVR, as shown in Figure 6a. This means we can measure the degree of finger motion including speed. Currently, wrist rotation detection has not yet been done. To monitor wrist rotation, we attached the sensor along the forearm, as shown in Figure 6b insets. When the test subject rotated the forearm from the neutral position to pronation, the radius moved markedly relative to the attached sensor, twisting the sensor and changing its resistance. Different forearm rotation speeds lead to different RVRs, as shown in Figure 6b.

A tiny pressure, such as that used when “clicking” a mouse or trackpad, can also be detected by the G/GL based sensor, as shown in Figure 6c. The sensor can also detect the movement of the Adam’s apple, such as when drinking water, as shown in Figure 6d. When the Adam’s apple moves up, the bent sensor loosens, resulting in lower resistance. When the Adam’s apple returns to the resting position, the sensor tenses again resulting in higher resistance.

## 4. Conclusions

We present here a novel, multifunctional and flexible G/GL based strain sensor which leverages the self-lubrication and viscosity properties of the graphene/glycerol solution. The sensitivity of these sensors can be controlled by adjusting the mass ratio of graphene in the G/GL solution. The developed strain sensor has a 1000% stretchability and maximum GF of 45.13. After a 10,000-cycle 200% strain test, the G/GL based sensors still have excellent dynamic performance. It can also detect small strains (1%, 5%, 10%) and different stretching rates (12.5%/s, 25%/s, 50%/s and 100%/s) due to the self-lubricating property of the glycerol in the G/GL solution. In addition to stretching, the G/GL sensors can also detect pressure (0–50 kPa) and twisting (0–180°). Finally, the strain sensors can be attached to human skin and can distinguish static and dynamic motion via changes in the RVR curves, making them suitable for measuring finger bending, wrist rotating, touching (“clicking”), and water drinking. Therefore, we believe that our multifunctional G/GL based sensors can provide excellent performance in stable sensing applications, especially in human motion detection.

## Figures and Tables

**Table 1:** Summary of performance results of recently reported stretchable strain sensors based on graphene or liquid conductors.

References	State of Sensing Layer	Materials	Stretchability (%)	Gauge Factor
this work	liquid	graphene/glycerin-Ecoflex	1000	45
[26]	liquid	reduced graphene oxide/DI-Ecoflex	400	31.6
[30]	liquid	ethylene glycol/NaCl-Ecoflex	250	<4
[31]	liquid	GaInSn liquid metal-PDMS(polydimethylsiloxane)	50	<2
[32]	liquid	NaCl solution-Ecoflex	50	
[33]	solid + liquid	graphene/ionic conductor−Ecoflex	300	25.2
[14]	solid	CNTs–PDMS	280	0.82
[34]	solid	CNTs–PDMS	45	35.75
[35]	solid	graphite-paper	±0.62	536.6
[36]	solid	graphite-poly(vinyl chloride)	±0.78	12
[7]	solid	AgNanoWires–PDMS	70	14
[37]	solid	CNTs–Ecoflex	150	1
[38]	solid	CBs(carbon blacks)–Ecoflex	400	3.8

## Supplementary Materials

The following are available online at https://www.mdpi.com/2079-4991/9/4/617/s1, Table S1: GFs at 100% strain of all sensors used to complete all the tests.
