# Supplementary material for: Graphene/Glycerin Solution-Based Multifunctional Stretchable Strain Sensor with Ultra-High Stretchability, Stability, and Sensitivity
Source: Nanomaterials (Basel). 2019 Apr 16;9(4):617. doi: 10.3390/nano9040617 (PMC6523101; doi:10.3390/nano9040617)
Supplement: Supplementary file 1 [file nanomaterials-09-00617-s001.pdf]

Supporting Information

Graphene/Glycerin Solution-Based Multifunctional Stretchable Strain Sensor with Ultra-High Stretchability, Stability, and Sensitivity

Zhenkun Qi, Hailiang Bian, Yi Yang, Nantian Nie and Fuliang Wang *

School of Mechanical and Electrical Engineering, Central South University, Changsha 410083, China; qizhenkun@csu.edu.cn (Z.Q.); csucmeebhl@csu.edu.cn (H.B.); youngwhyi_csu@163.com (Y.Y.); nienantian@csu.edu.cn (N.N.)

* Correspondence: wangfuliang@csu.edu.cn, Tel.: +86-139-7514-5436

Table S1. GFs at 100% strain of all sensors used to complete all the tests.

NO.	GF	test
sensor 1	3.4	Effect of graphene concentration in G/GL solution
sensor 2	4.3	1000% stretchability
sensor 3	3.9	different strain speed
sensor 4	4.5	
sensor 5	3.8	
sensor 6	4.6	response time
sensor 7	4.0	
sensor 8	4.1	
sensor 9	4.3	long time cyclic test
